# The impact of diabetes on tuberculosis treatment outcomes: A systematic review

**DOI:** 10.1186/1741-7015-9-81

**Published:** 2011-07-01

**Authors:** Meghan A Baker, Anthony D Harries, Christie Y Jeon, Jessica E Hart, Anil Kapur, Knut Lönnroth, Salah-Eddine Ottmani, Sunali D Goonesekera, Megan B Murray

**Affiliations:** 1 Division of Infectious Diseases, Massachusetts General Hospital, Boston, MA, USA; 2 Department of Epidemiology, Harvard School of Public Health, Boston, MA, USA; 3 International Union Against Tuberculosis and Lung Disease, Paris, France; 4 London School of Hygiene and Tropical Medicine, Keppel Street, London, UK; 5 Center for Infectious Disease Epidemiologic Research, Columbia University, New York, NY, USA; 6 The Warren Alpert Medical School of Brown University, Providence, RI, USA; 7 World Diabetes Foundation, Lyngby, Denmark; 8 Stop-TB Department, World Health Organization, Geneva, Switzerland; 9 Division of Global Health Equity, Brigham & Women's Hospital, Boston, MA, USA; 10 Department of Epidemiology, Harvard School of Public Health, Boston, MA, USA

## Abstract

**Background:**

Multiple studies of tuberculosis treatment have indicated that patients with diabetes mellitus may experience poor outcomes.

We performed a systematic review and meta-analysis to quantitatively summarize evidence for the impact of diabetes on tuberculosis outcomes.

**Methods:**

We searched PubMed, EMBASE and the World Health Organization Regional Indexes from 1 January 1980 to 31 December 2010 and references of relevant articles for reports of observational studies that included people with diabetes treated for tuberculosis. We reviewed the full text of 742 papers and included 33 studies of which 9 reported culture conversion at two to three months, 12 reported the combined outcome of failure and death, 23 reported death, 4 reported death adjusted for age and other potential confounding factors, 5 reported relapse, and 4 reported drug resistant recurrent tuberculosis.

**Results:**

Diabetes is associated with an increased risk of failure and death during tuberculosis treatment. Patients with diabetes have a risk ratio (RR) for the combined outcome of failure and death of 1.69 (95% CI, 1.36 to 2.12). The RR of death during tuberculosis treatment among the 23 unadjusted studies is 1.89 (95% CI, 1.52 to 2.36), and this increased to an effect estimate of 4.95 (95% CI, 2.69 to 9.10) among the 4 studies that adjusted for age and other potential confounding factors. Diabetes is also associated with an increased risk of relapse (RR, 3.89; 95% CI, 2.43 to 6.23). We did not find evidence for an increased risk of tuberculosis recurrence with drug resistant strains among people with diabetes. The studies assessing sputum culture conversion after two to three months of tuberculosis therapy were heterogeneous with relative risks that ranged from 0.79 to 3.25.

**Conclusions:**

Diabetes increases the risk of failure and death combined, death, and relapse among patients with tuberculosis. This study highlights a need for increased attention to treatment of tuberculosis in people with diabetes, which may include testing for suspected diabetes, improved glucose control, and increased clinical and therapeutic monitoring.

## Background

Tuberculosis (TB) remains a major source of morbidity and mortality throughout the world; one-third of the world's population is estimated to be infected with *Mycobacterium tuberculosis*, approximately nine million people develop the disease each year, and almost two million die annually from the disease [1,2]. Epidemiological studies have elucidated an association between diabetes mellitus (DM) and the development of TB disease [3-7]. According to a recent systematic review, among cohort studies, people with DM had approximately three times the risk of developing TB disease as people without [4]. The global burden of DM is rising; the prevalence is estimated to reach 438 million by 2030, and more than 80% of the adult cases will be in newly developed or developing countries [8]. The convergence of these two epidemics may lead to an increased incidence of TB disease, especially in low and middle income countries with increasing numbers of people with DM and prevalent TB disease [5,9]. For example, in areas such as the border population of South Texas and Mexico with a high prevalence of DM, self-reported DM is the most common risk factor associated with TB [10].

Among patients afflicted with both TB and DM, diabetes is reported to be associated with poor TB treatment outcomes [7,11,12]; however, a systematic analysis to both clarify and quantify the association between DM and TB outcomes, including persistence of sputum culture positivity, failure, death and relapse, has not been performed. Given the increasing burden of TB patients with DM globally, an association between DM and TB outcomes would suggest that diabetes could increase the number of persons infected by a source case and the number of patients needing anti-TB retreatment regimens. Further clarification and quantification of the association between DM and these outcomes will inform public health measures, and we conducted a systematic review and meta-analysis to this end.

## Methods

We conducted this study according to the Meta-analysis of Observational Studies in Epidemiology (MOOSE) guidelines [13].

### Data sources and searches

We searched the PubMed via the NCBI Entrez system http://www.ncbi.nlm.nih.gov/entrez/query.fcgi, the EMBASE via Ovid http://www.ovid.com, and the World Health Organization Regional Indexes (AIM (AFRO), LILACS (AMRO/PAHO), IMEMR (EMRO), IMSEAR (SEARO), WPRIM (WPRO)) http://www.globalhealthlibrary.net/php/index.php from 1 January 1980 to 31 December 2010 for studies of the association between DM and TB disease outcomes. We also searched bibliographies of identified reports for additional references. Our search strategy is defined in Panel 1.

#### Panel 1. Search strategy for studies on the association between DM and TB outcomes

PubMed:

MeSH Terms:

1. Tuberculosis

2. "Diabetes mellitus"

Text Terms:

3. Outcome(s) OR Treatment(s)

4. Risk factor(s)

5. Tuberculosis

6. "Diabetes mellitus"

Search Strings (all inclusive)

a) 1 AND 2

b) 1 AND 3 AND 4

c) 5 AND 6 (for the year preceding 12/10 in which articles may not have been assigned MeSH terms)

EMBASE and World Health Organization Regional Indexes:

1. Tuberculosis, major subject

2. "Diabetes mellitus"

3. Outcome(s) OR Treatment(s)

4. Risk factor(s)

Search Strings (all inclusive)

a) 1 AND 2

b) 1 AND 3 AND 4

### Study selection

We included studies regardless of language. We compared sources to exclude duplicate references and contacted authors for data that were not available in publications and abstracts. Studies were included if they met the following criteria: 1) They were peer-reviewed reports of studies involving human participants receiving pharmacologic anti-mycobacterial treatment for TB disease. 2) They provided or permitted the computation of an effect estimate of the relationship between DM and at least one of the following five TB treatment outcomes: proportion of treated patients who experienced culture conversion at two to three months, the combined outcome of treatment failure and death, death, relapse, or recurrence with drug-resistant (DR) TB. Treatment failure was defined as sputum smear or culture positivity at five months or later during treatment [14,15]. We combined failure and death into a single endpoint that represents poor outcomes. Death was defined as death for any reason during the course of treatment [14-16]. Relapse was defined as bacteriologically positive TB disease that occurred after a patient was considered to have completed treatment or to have been cured [14-16]. Recurrence was defined as TB disease that occurred in a patient with a history of prior treatment for TB. 3) They defined DM as any of the following: baseline diagnosis by self-report, medical records, fasting blood glucose (FBG) ≥ 126 mg/dL or ≥ 140 mg/dL (to reflect the present and past American Diabetes Association Guidelines and World Health Organization (WHO) recommendations for the diagnosis of DM [17,18]), non-FBG ≥ 200 mg/dL, or treatment with oral hypoglycemic medications or insulin.

We excluded the following: citations without abstracts; anonymous reports; duplicate studies; case reports or studies which did not compare outcomes among people with DM to people without DM; reviews; studies that did not report outcomes in adults; studies that examined the reverse association of the impact of TB disease on DM or diagnosed DM during TB treatment; studies where the majority of treatment took place prior to 1980, in order to incorporate short course chemotherapy and assess consistent TB treatment regimens [15]; studies after 1995 that did not adjust for human immunodeficiency virus (HIV) status if study participants came from countries with a prevalence of HIV among adults (15 to 49 years) of > 5%; studies that did not report at least one of the TB outcomes listed above; studies that did not follow patients for the duration of TB treatment for the outcome of failure and death; studies that assessed the risk of relapse that did not follow patients from the first episode of TB; studies in which people with DM received different anti-TB treatment regimens than people without DM; and studies that either did not provide effect estimates in odds ratios (ORs), rate ratios, hazard ratios (HRs), or RRs or did not allow for the computation of these values.

### Data extraction and quality assessment

For every study that met our eligibility criteria, two investigators (MB and JH or CJ) independently collected detailed information on the year, country, study design, study population, type of TB outcome, diagnosis of DM and TB, adjustment for age, HIV and other potential confounders, proportion of treated patients who experienced the outcomes of interest, effect sizes, and 95% confidence intervals. Differences were resolved by discussion and consensus. For non-English language papers, bilingual translators trained in medicine or public health helped classify studies and extract data with MB and JH.

TB death and relapse are relatively rare events; therefore, we assumed that ORs, RRs, and HRs provided a similar risk estimate, and we reported them as a common effect estimate in the death and relapse analyses [19].

### Data synthesis and analysis

We performed separate analyses for each of the outcomes and assessed heterogeneity of effect estimates using the Cochran Q test for heterogeneity and the I^2 ^statistic described by Higgins *et al. *[20,21]. The 95% confidence intervals (CIs) for the I^2 ^were calculated using the test-based methods [21]. We performed meta-analysis to compute a summary estimate only for those studies that did not show significant heterogeneity, defined as I^2 ^< 50% [21]. We decided *a priori *to use the random effects model and weighting method according to the method described by DerSimonian and Laird [22] as we expected the true effect of DM on TB outcomes to vary and because it would yield conservative 95% CIs.

We addressed potential causes of heterogeneity and the impact of study quality for the outcome analyses with an I^2 ^> 10%. We compared pooled effect estimates for subgroups categorized by background TB incidence and pulmonary versus other types of TB, and by the following quality-associated variables: time of assessment of DM in relation to TB diagnosis, exposure classification (self-report, medical records and DM medications versus laboratory tests), loss to follow-up, and the use of survival analysis. We considered studies to be of higher quality if they specified that patients were diagnosed with DM prior to TB diagnosis, if DM was diagnosed based on medical records, self-report or use of DM medications (blood glucose measurements at the time of TB diagnosis may overestimate DM, as TB disease is associated with increased blood glucose levels [23,24]), if studies adjusted for at least age, if they reported loss to follow-up through default and transfer out of less than 10% of the cohort, and if they estimated a HR using survival analysis. We regressed study-specific log-transformed RRs by the variables representing the study characteristics, weighting the studies by the inverse of the sum of within-study and between-study variance for all studies within the comparison. Coefficients of meta-regression represent differences in log-transformed RRs between the subgroups. We tested the significance of these coefficients by Student's *t-*test, and the significance was set at *P *< 0.05. We performed a separate analysis for studies assessing the outcome of death that adjusted for age and other confounding factors.

We assessed publication bias using Begg's and Egger's tests [25,26] and by visual inspection for asymmetry of a plot of the natural logarithms of the effect estimates against the standard errors [25]. Statistical procedures were performed using STATA version 10, Texas [27].

## Results

**Figure 1:**
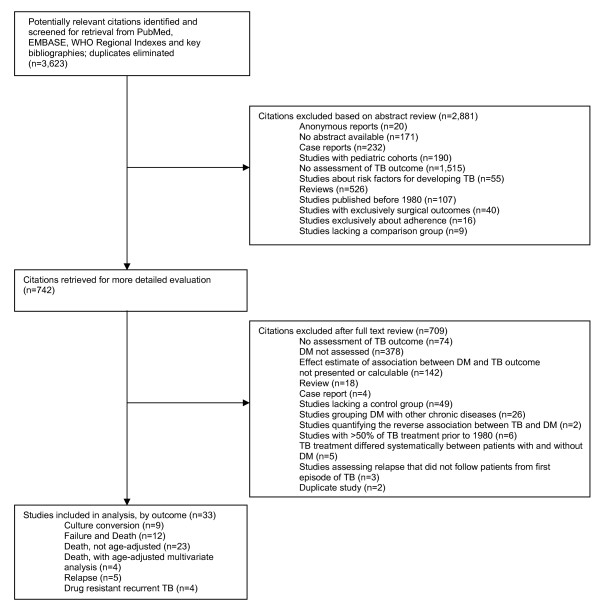
**The literature search for studies on the association between DM and TB outcomes**.

We identified and screened 3,623 papers by titles and abstracts, including 2,841 papers in English and 782 papers in other languages. We excluded 2,881 papers, because they did not study TB outcomes, studied exclusively surgical interventions, lacked a comparison group, were studies conducted exclusively among children, were published before 1980, lacked an abstract, or were case reports, reviews, or anonymous reports (Figure 1). The full texts of the remaining 742 papers were analyzed and on the basis of that review, we excluded 709 articles because they did not present an effect estimate or provide data from which an effect estimate could be calculated (142), they did not assess DM (378), they did not assess the TB outcomes defined in the methods (74), they lacked a comparison group without DM (49), they grouped DM with other chronic diseases (26), the treatment regimen differed between the population with DM and without DM (5), they were reviews, case reports, or duplicate studies (24), the majority of treatment took place before 1980 (6), they did not follow patients from the first episode of TB for the outcome of relapse (3), or they measured the reverse association between DM and TB or diagnosed DM after TB diagnosis (2). No studies conducted in countries with a high prevalence of HIV were excluded on the basis of not adjusting for HIV status. We contacted 21 authors for further information and clarification and obtained additional data from 7 of these. We included 33 studies of which 9 reported culture conversion at two to three months, 12 reported the combined outcome of failure and death, 23 reported death, 4 reported death adjusted for age and other potential confounding factors, 5 reported relapse, and 4 reported recurrence with drug resistant tuberculosis (Table 1) [3,11,12,28-57]. The included studies were written in English (25), Japanese (4), French (2), and Spanish (2).

**Table 1:** Characteristics of included studies for the association between DM and TB outcomes

						Outcomes						
							
Study	Type of study	Country	Type of TB	Total n	Population with DM n	Sputum Culture Conversion 2-3 months	Failure And Death	Death	Adjusted Variables for Death Outcome	Relapse	DR Recurrence	DM Definition
Alisjahbana [11]	Prospective cohort	Indonesia	Pulmonary TB	634	94	√	√	√				2 measurements of FBG > 126 mg/dL
Ambrosetti [28]	Prospective cohort	Italy	Undifferentiated TB	778	32		√	√				Medical records
Ambrosetti [29]	Prospective cohort	Italy	Undifferentiated TB	838	50		√	√				Medical records
Ambrosetti [30]	Prospective cohort	Italy	Undifferentiated TB	715	40		√	√				Medical records
Anunnatsiri [31]	Retrospective cohort	Thailand	Pulmonary TB	226	117		√					Medical records
Banu Rekha [32]	Retrospective analysis of 3 concurrent studies	India	Pulmonary TB	190	92	√						Medical records, FBG
Bashar [33]	Retrospective case-control	USA	Undifferentiated TB	155	50			√				Medical records
Blanco [34]	Retrospective cohort	Canary Islands, Spain	Pulmonary TB	98	14	√						Medical records
Centis [35]	Prospective cohort	Italy	Undifferentiated TB	1,162	56		√	√				Medical records
Centis [36]	Prospective cohort	Italy	Undifferentiated TB	906	40		√	√				Medical records
Chiang [37]	Retrospective cohort	Taiwan	Pulmonary TB	1,127	241		√	√				Medical records
Dooley [12]	Retrospective cohort	USA	Undifferentiated TB	297	42	√		√	Age, HIV, weight, foreign birth			Medical records, non-FBG > 200 mg/dL, DM medications
Fielder [38]	Retrospective cohort	USA	Pulmonary TB	174	22			√	Age			Medical records
Fisher-Hoch [39]	Retrospective cohort	Mexico & USA	Undifferentiated TB	2,878	688			√			√	Self report
Guler [40]	Retrospective cohort	Turkey	Pulmonary TB	306	44	√						Medical records
Hara [41]	Retrospective cohort	Japan	Pulmonary TB	624	112	√						Medical records
Hasibi [42]	Retrospective cohort	Iran	Disseminated TB	50	6			√				Medical records
Ito [43]	Retrospective cohort	Japan	Undifferentiated TB	109	16						√	Medical records
Kitahara [44]	Retrospective cohort	Japan	Pulmonary TB	520	71	√		√				Medical records
Kourbatova [45]	Retrospective case-control	Russia	Undifferentiated TB	460	20			√				Medical records
Maalej [46]	Retrospective case-control	Tunisia	Pulmonary TB	142	60			√		√		Medical records
Mboussa [47]	Retrospective cohort	Republic of the Congo	Pulmonary TB	132	32		√	√		√		2 measurements of FBG ≥ 126 mg/dL
Oursler [48]	Retrospective cohort	USA	Pulmonary TB	139	18			√	Age, HIV, renal, COPD			Medical records
Pina [49]	Retrospective cohort	Spain	Undifferentiated TB	1,511	73			√				Medical records
Ponce-De-Leon [3]	Prospective cohort	Mexico	Pulmonary TB	581	172		√	√				Medical records (FBG ≥ 126 mg/dL, non-FBG ≥ 200 mg/dL sensitivity analysis)
Singla [50]	Retrospective cohort	Saudi Arabia	Pulmonary TB	692	187		√	√		√		2 measurements of FBG > 140 mg/dL
Subhash [51]	Retrospective cohort	India	Undifferentiated TB	361	72						√	FBG > 140 mg/dL, medical records and DM medication or diet
Tatar [52]	Retrospective cohort	Turkey	Undifferentiated TB	156	78	√		√				Medical records
Vasankari [53]	Retrospective cohort	Finland	Pulmonary TB	629	92			√				Treatment with DM medications
Wada [54]	Retrospective cohort	Japan	Pulmonary TB	726	143	√				√		Medical records
Wang [55]	Retrospective cohort	Taiwan	Pulmonary TB	453	75						√	Medical records
Wang [56]	Retrospective cohort	Taiwan	Pulmonary TB	217	74		√	√	Age, sex			Medical records and DM medication or FBG > 126 mg/dL
Zhang [57]	Retrospective Cohort	China	Pulmonary TB	2,141	203					√		FBG ≥ 126 mg/dL

### Sputum culture conversion at two to three months

**Figure 2:**
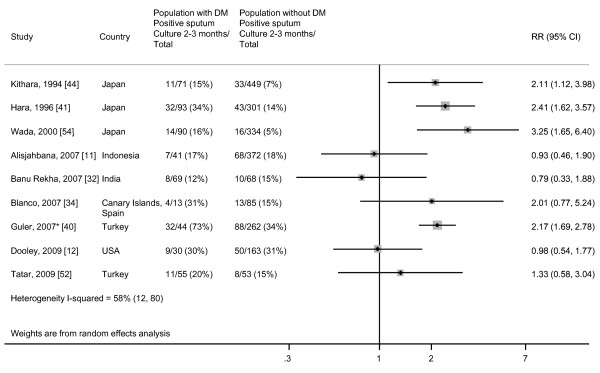
**Risk of remaining sputum culture positive for TB patients with DM compared with TB patients without DM**. Size of the square is proportional to the precision of the study-specific effect estimates, and the bars indicate the corresponding 95% CIs. *The RR for Guler *et al. *[40] was calculated using the OR, CI and total number of patients with and without DM provided in the paper.

We found substantial heterogeneity of effect estimates among the nine studies that assessed the risk of remaining sputum culture positive after two to three months of anti-TB therapy comparing patients with and without DM (Figure 2). Relative risks ranged from 0.79 to 3.25, and between-study variance accounted for 58% of the total variance among studies. Because of this heterogeneity, we do not report a summary estimate. We found no evidence for publication bias by either Begg's test (*P *= 0.30) or Egger's test (*P *= 0.27) (Additional file 1). Among the three studies that reported RRs of < 1 for the risk of sputum culture positivity at two to three months, one reported a significant difference in sputum culture conversion at six months (unadjusted OR 2.69 (95% CI, 1.01 to 7.14), adjusted OR 7.65 (95% CI, 1.89 to 30.95)) [11], while another found a trend toward increased time to sputum culture conversion among patients with diabetes (*P *= 0.09) [12].

The sensitivity analysis presented in Table 2 shows that the estimated risk of remaining sputum culture positive at two to three months was greater in studies that used medical records, patient report and medication history to classify patients with diabetes, rather than laboratory tests conducted at the initiation of treatment (RR 2.22 (95% CI, 1.85 to 2.66) and RR 0.92 (95% CI, 0.62 to 1.38) respectively) (meta-regression *P *< 0.01). None of the studies explicitly stated that DM was diagnosed prior to TB.

**Table 2:** Sensitivity analysis to identify sources of heterogeneity in the association between DM and TB

TB Outcome	Variables	Study Characteristics (No. of studies)	Summary effect estimates	95% CI	I^2^	95% CI I^2^	P Value Heterogeneity	P Value Meta- regression
Sputum culture conversion at 2-3 months	Background TB incidence	≥ 100,000 (2)	0.87	0.50-1.51	0%	NA	0.77	0.53
		≥ 10,000 and < 100,000 (6)	2.22	1.85-2.66	0%	0%-75%	0.70	
		< 10,000 (1)	0.98	0.54-1.77	NA	NA	NA	
	Type of TB	Pulmonary (7)	1.91	1.41-2.59	52%	0%-79%	0.05	0.18
		Pulmonary and extrapulmonary (2)	1.08	0.67-1.76	0%	NA	0.56	
	DM diagnosis	Patient or medical report (6)	2.22	1.85-2.66	0%	0%-75%	0.71	< 0.01
		Laboratory test (3)	0.92	0.62-1.38	0%	0%-90%	0.92	
	Loss to follow-up	< 10% (1)	1.33	0.58-3.04	NA	NA	NA	0.84
		≥ 10% (2)	1.75	0.51-5.95	84%	NA	0.01	
Failure and Death	Background TB incidence	≥ 100,000 (3)	2.11	1.26-3.53	20%	0%-92%	0.29	0.63
		≥ 10,000 and < 100,000 (6)	1.49	1.23-1.80	0%	0%-75%	0.51	
		< 10,000 (3)	1.80	0.63-5.13	55%	0%-87%	0.11	
	Type of TB	Pulmonary (7)	1.62	1.26-2.07	26%	0%-68%	0.23	0.49
		Pulmonary and extrapulmonary (5)	2.08	1.27-3.42	6%	0%-80%	0.38	
	DM diagnosis	Patient or medical report (8)	1.51	1.25-1.82	0%	0%-68%	0.49	0.10
		Laboratory diagnosis (4)	1.97	1.12-3.46	42%	0%-80%	0.16	
	Loss to follow-up	< 10% (4)	1.72	1.25-2.37	52%	0%-84%	0.10	0.98
		≥ 10% (8)	1.77	1.21-2.59	3%	0%-69%	0.41	
Death	Background TB incidence	≥ 100,000 (3)	2.63	0.86-8.02	62%	0%-89%	0.07	0.98
		≥ 10,000 and < 100,000 (13)	1.62	1.33-1.97	8%	0%-60%	0.37	
		< 10,000 (7)	1.95	1.12-3.40	74%	45%-88%	< 0.01	
	Type of TB	Pulmonary (11)	1.97	1.46-2.65	52%	5%-76%	0.02	0.59
		Pulmonary and extrapulmonary (12)	1.84	1.28-2.64	41%	0%-70%	0.07	
	DM diagnosis	Patient or medical report (18)	1.82	1.42-2.32	50%	15%-71%	< 0.01	0.33
		Laboratory diagnosis (5)	2.37	1.49-3.78	1%	0%-79%	0.40	
	Loss to follow-up	< 10% (9)	1.43	1.19-1.72	16%	0%-58%	0.30	0.12
		≥ 10% (7)	2.22	1.24-3.98	21%	0%-64%	0.27	
	Survival analysis	Survival analysis (1)	4.8	2.0-11.6	NA	NA	NA	0.18
		Alternative analysis (22)	1.81	1.45-2.26	42%	4%-65%	0.02	

### Failure and death

**Figure 3:**
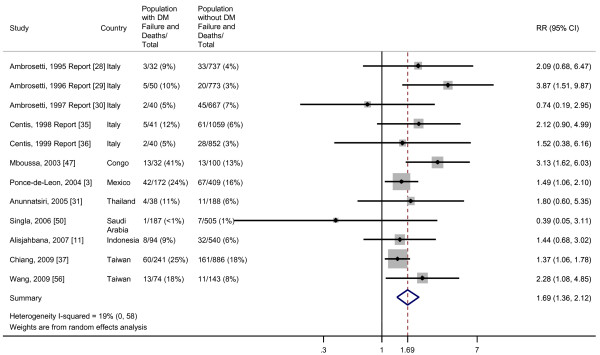
**Risk of failure/death for TB patients with DM compared with TB patients without DM**. Size of the square is proportional to the precision of the study-specific effect estimates, and the bars indicate the corresponding 95% CIs. The diamond is centered on the summary RR of the observational studies, and the width indicates the corresponding 95% CI.

The pooled RR of the combined outcome, failure and death, among the 12 studies that included both outcomes was 1.69 (95% CI, 1.36 to 2.12). Between-study variance accounted for 19% of the total variance (Figure 3). Although Egger's test suggested publication bias (*P *= 0.01), Begg's test was not significant (*P *= 0.49) (Additional file 2). The sensitivity analysis did not include several of the quality associated strata, because none of the reviewed studies explicitly stated that the DM diagnosis predated the TB diagnosis, and none performed a survival analysis. As shown in Table 2 although some of the variability among the studies is explained by the variables included, substantial heterogeneity remains after the meta-regression.

### Death

**Figure 4:**
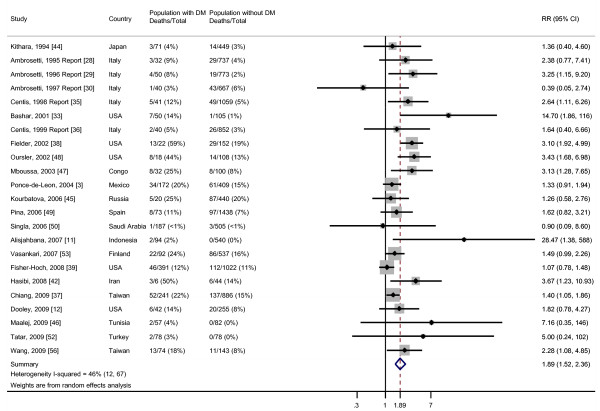
**Risk of death for TB patients with DM compared with TB patients without DM**. Size of the square is proportional to the precision of the study-specific effect estimates, and the bars indicate the corresponding 95% CIs. The diamond is centered on the summary RR of the observational studies, and the width indicates the corresponding 95% CI.

Among the 23 studies that compared the risk of death during TB treatment in patients with DM versus patients without DM, we found moderate heterogeneity of effect estimates with between study variance accounting for 46% of the total variance (Figure 4). The pooled RR from the random effects analysis was 1.89 (95% CI, 1.52 to 2.36). Although Egger's test suggested publication bias (*P *= 0.01), Begg's test was not significant (*P *= 0.19) (Additional file 3). The one study that evaluated the effect estimate using survival analysis found a HR of 4.8 (95% CI, 2.0 to 11.6) [48]. None of the studies explicitly stated that DM was diagnosed prior to TB.

**Figure 5:**
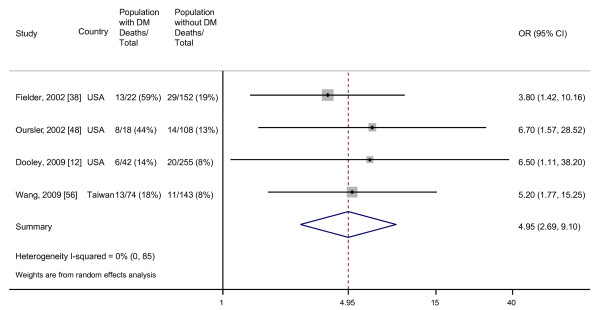
**Adjusted odds of death for TB patients with DM compared with TB patients without DM**. Size of the square is proportional to the precision of the study-specific effect estimates, and the bars indicate the corresponding 95% CIs. The diamond is centered on the summary OR of the observational studies, and the width indicates the corresponding 95% CI.

When we restricted the analysis to those four studies which adjusted for age and other potential confounders, we did not find heterogeneity among the effect estimates despite the fact that each study controlled for a different set of confounders. The random effects pooled OR was 4.95 (95% CI, 2.69 to 9.10) (Figure 5). There was no evidence for publication bias by Begg's test (*P *= 0.17) or Egger's test (*P *= 0.18) (Additional file 4).

### Relapse

**Figure 6:**
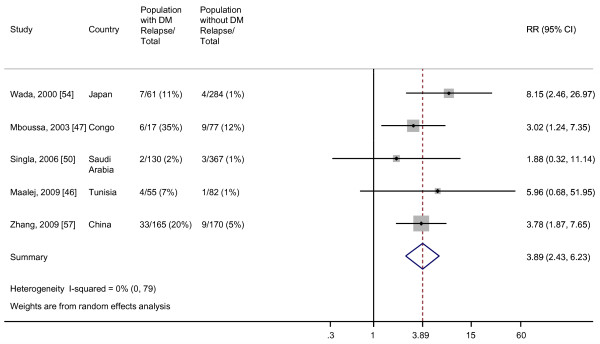
**Risk of TB relapse for TB patients with DM compared with TB patients without DM**. Size of the square is proportional to the precision of the study-specific effect estimates, and the bars indicate the corresponding 95% CIs. The diamond is centered on the summary RR of the observational studies, and the width indicates the corresponding 95% CI.

Among the five studies that assessed the risk of TB relapse, the random effects pooled RR was 3.89 (95% CI, 2.43 to 6.23) for relapse after TB cure or treatment completion among patients with DM versus patients without DM (Figure 6). There was no evidence for heterogeneity among the studies that assessed this outcome and no evidence for publication bias by Begg's test (*P *= 1.00) or Egger's test (*P *= 0.81) (Additional file 5).

### Drug resistant recurrent disease

**Figure 7:**
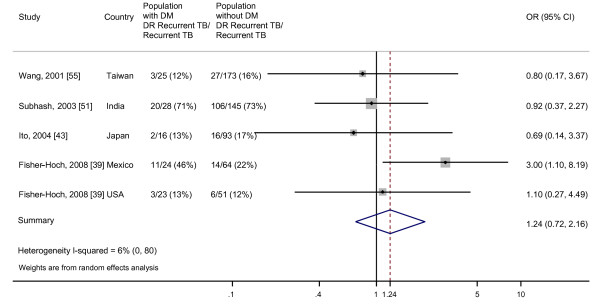
**Odds of recurrent TB that is DR, comparing patients with DM to patients without DM**. Size of the square is proportional to the precision of the study-specific effect estimates, and the bars indicate the corresponding 95% CIs. The diamond is centered on the summary OR of the observational studies, and the width indicates the corresponding 95% CI.

The random effects pooled OR was 1.24 (95% CI, 0.72 to 2.16) for the four studies that assessed the odds of developing recurrent TB that is DR (Figure 7). Studies were not heterogeneous, as between study variance accounted for 6% of the total variance among the studies included in this analysis. We did not find evidence for publication bias by Begg's test (*P *= 0.62) or Egger's test (*P *= 0.76) (Additional file 6).

## Discussion

This systematic review of the impact of DM on outcomes of TB treatment determines that DM increases the risk of the combined outcome of failure and death, death, and relapse. Notably, the pooled effect estimate for death among studies that adjusted for age and other confounding factors was found to be higher than the pooled effect estimate among the unadjusted studies. This finding not only suggests that patients with DM receiving TB therapy are at risk for poor outcomes, but that outcome studies that do not control for appropriate confounders may underestimate the negative impact of DM in TB patients. Future studies of the impact of DM on TB outcomes should be designed to minimize the potential bias due to confounding factors such as age and HIV.

The results of the studies analyzing culture positivity at two to three months after initiation of TB treatment were heterogeneous with five of the nine studies reporting an RR of greater than two and three reporting an RR of less than one. Two of those three papers reported delay in sputum culture conversion in persons with DM at some point during the course of treatment [11,12]. Thus, all but one of the papers included in this analysis reported delay in sputum culture conversion, although this outcome occurred at different times.

The effect of DM on both the combined outcome of failure and death and death alone in studies that did not adjust for age and other confounding factors was relatively modest; however, the effect on death not only persisted, but increased among those studies that adjusted for potential confounders. An explanation for the higher risk observed in the studies that adjusted for confounding factors may be that patients who die during TB treatment have other strong risk factors for death such as HIV or co-morbidities that tend to reduce the apparent impact of DM in the unadjusted analyses. However, because the studies that adjusted for confounders were all performed in industrialized countries where TB mortality rates tend to be high [58], it is difficult to generalize these results to higher burden settings.

We expect that the risks of the combined outcome, failure and death, and death alone are underestimated due to loss to follow-up. The patients who default or are transferred out are not followed for the entire treatment period, and their final outcome is therefore not observed. In the one study that used survival analysis to adjust for this loss of follow-up, the HRs were higher than the pooled effect estimates in both the univariate and adjusted analyses [48]. Since the rate of death is higher among persons with DM than those without at baseline, the increased death rate may reflect that risk rather than indicate a higher rate of TB associated mortality among those with DM [59]. However, the differential age specific mortality experienced by persons with DM probably does not account for the odds ratio of death of almost 5 in the adjusted analysis.

Although we found evidence for publication bias in the analysis of the combined outcome, failure and death, and the unadjusted analysis of death when we used Egger's test, this was not confirmed with Begg's test, a method that is less susceptible to false positive results [60,61], nor was it found in the analysis of death restricted to studies that adjusted for age and other confounders.

Patients with DM were four times more likely to develop a relapse of TB disease than patients without DM. Considering the fact that these individuals were considered cured or treatment complete, the patients could have relapsed through one of two possible routes: they may have been cured but experienced a recurrence of the former infection, or they may have been re-infected with a new strain of TB. The increased risk of recurrent disease in either of these scenarios is consistent with prior evidence suggesting that those with DM are at increased risk of developing TB disease [3-6]. Furthermore, health facility exposure to TB, an important contributor to the total TB infection risk in people living with HIV [62], may contribute to the risk of re-infection in patients with DM due to repeated attendance at health facilities for diabetes management.

The results in this systematic review may underestimate the risk of relapse, because patients with DM are more likely to die during a first course of TB therapy and in the time period prior to a diagnosis of relapse [63]. Thus, patients with DM may be less likely to have recurrent TB than patients without DM because of loss to competing risks. This methodological consideration suggests that the appropriate study design to assess relapse is survival analysis, and only one of the studies reported here used that approach [54]. Other limitations of this analysis included the small numbers of relapses that occurred and lack of adjustment for confounding factors.

We found no evidence that DM increased the risk of recurrent disease with DR TB, despite the greater risk for TB disease and impaired cell mediated immunity [64-68]. This finding is consistent with data from a recent pharmacological study that reported therapeutic levels of rifampicin, pyrazinamide and ethambutol in patients with DM who received standard dosing during the intensive phase of TB treatment [69]. However, if the effect of DM is relatively small, the four studies with only 208 cases of DR recurrent disease may not have had sufficient power to detect an association. Furthermore, these studies did not adjust for potential confounding factors such as HIV or frequency of medical care during TB treatment.

The increased risks of failure, death during TB treatment, and relapse among patients with DM are consistent with data from mouse models and human studies that show that DM impairs cell-mediated immunity [64-68]. Furthermore, a study by Restrepo *et al. *determines that poor diabetic control, as measured by glycosylated hemoglobin level, affects *in vitro *innate and type 1 cytokine responses [70]. We speculate that poor diabetes control, possibly exacerbated by TB disease, may be an important contributing factor to case fatality and relapse.

This study highlights the perils of using observational studies for a meta-analysis. Although meta-analyses of observational studies are frequently faulted for finding false statistically significant associations by combining small studies affected by confounding [71], we found evidence that an association may also be diminished by confounding or bias resulting from study design. Misclassification of the diagnosis of DM may have also diminished the association between DM and TB outcome. Since glucose levels are transiently increased in the setting of active TB [23,24], and the studies did not specify that the diagnosis of DM must precede that of TB, some patients diagnosed with DM may have been experiencing only a transient episode of hyperglycemia. The systematic review highlights the need for large-scale prospective studies with appropriate study design, prospective diagnoses of diabetes, control for confounding factors, and clear TB outcomes to further clarify the strength of the associations.

## Conclusions

This study reports that diabetes is associated with an increased risk of the combined endpoint of failure and death, death during TB treatment, and relapse. It is the first study that we are aware of that quantifies the associations based on a systematic review of the literature. The implications of the negative impact of DM on TB outcomes include poor individual outcomes, increased risk of secondary transmission, and increased incidence of TB disease. Considering the increasing burden of DM, particularly in areas with highly prevalent TB, TB control programs will need to expand efforts to focus on treatment and monitoring of patients with DM and TB disease.

## Abbreviations

CI: confidence interval; DM: diabetes mellitus; DR: drug-resistant; FBG: fasting blood glucose; HIV: human immunodeficiency virus; HR: hazard ratio; MOOSE: Meta-analysis of Observational Studies in Epidemiology; OR: odds ratio; RR: risk ratio; TB: tuberculosis; WHO: World Health Organization.

## Competing interests

The authors declare that they have no competing interests.

## Authors' contributions

MB participated in the design, literature search, analysis and drafting of the manuscript. AH participated in the conception, design, analysis and drafting of the manuscript. CJ participated in the design, data extraction and analysis. JH participated in the literature search and data extraction. AK, KL and SO conceived of the study and participated in the writing of the manuscript. SG participated in the literature search and data extraction. MM participated in the design, analysis, and drafting of the manuscript. All authors read and approved the final manuscript.

## Pre-publication history

The pre-publication history for this paper can be accessed here:

http://www.biomedcentral.com/1741-7015/9/81/prepub

## Supplementary Material

Additional file 1 **Additional file 1.ppt**. Begg's funnel plot with pseudo 95% confidence limits for all studies with sputum cultures at two to three months. Click here for file

Additional file 2 **Additional file 2.ppt**. Begg's funnel plot with pseudo 95% confidence limits for all studies with the combined outcome of failure and death. Click here for file

Additional file 3 **Additional file 3.ppt**. Begg's funnel plot with pseudo 95% confidence limits for all studies with the outcome of death. Click here for file

Additional file 4 **Additional file 4.ppt**. Begg's funnel plot with pseudo 95% confidence limits for all studies with the outcome of death adjusted for age and other confounding factors. Click here for file

Additional file 5 **Additional file 5.ppt**. Begg's funnel plot with pseudo 95% confidence limits for all studies with TB relapse. Click here for file

Additional file 6 **Additional file 6.ppt**. Begg's funnel plot with pseudo 95% confidence limits for all studies with recurrent TB that is DR. Click here for file
