# Supplementary figures and images for: The impact of diabetes on tuberculosis treatment outcomes: A systematic review
Source: BMC Med. 2011 Jul 1;9:81. doi: 10.1186/1741-7015-9-81 (PMC3155828; doi:10.1186/1741-7015-9-81)

## Slide 1
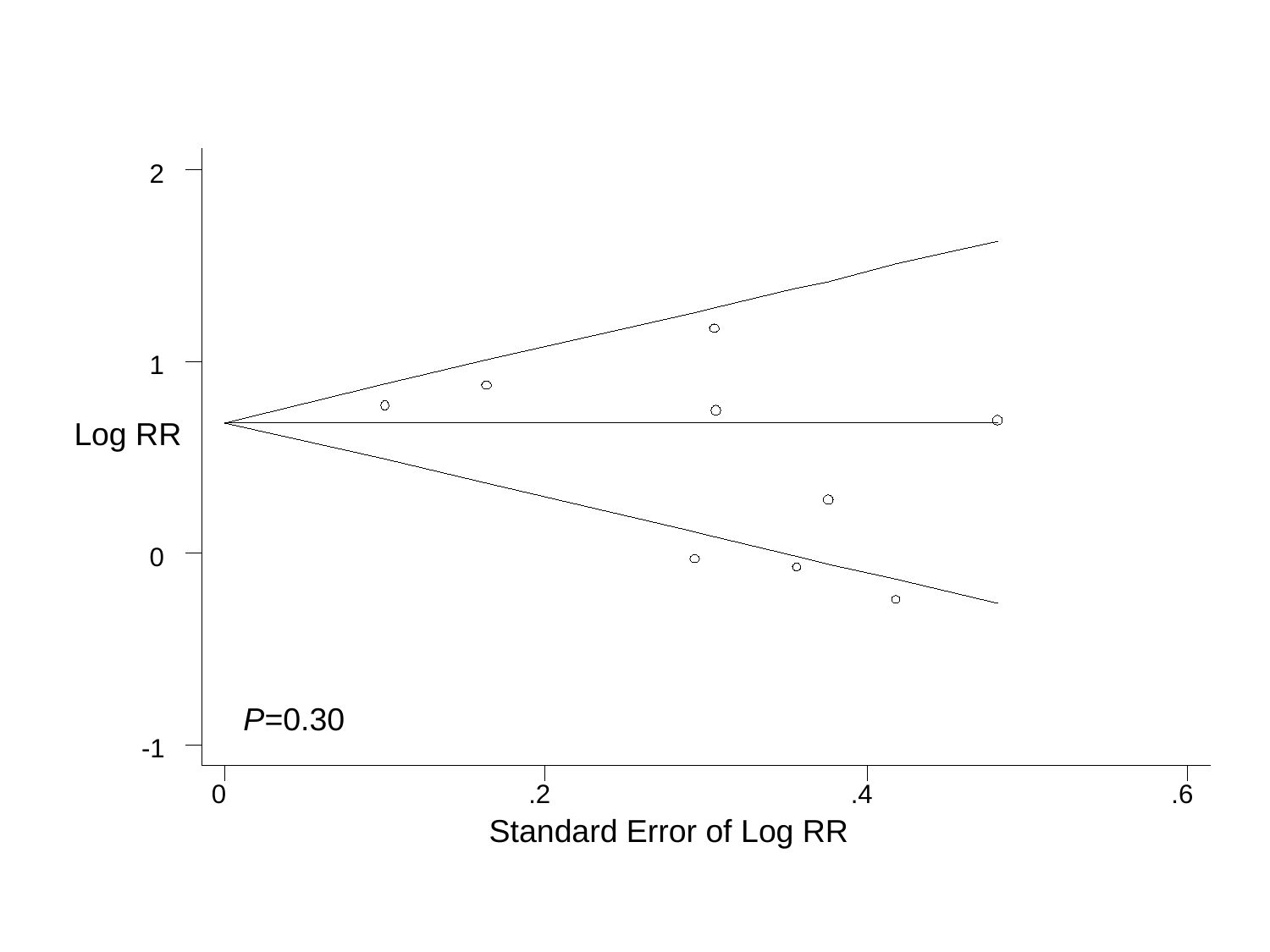

2
1
0
-1
0
.2
.4
.6
Log RR
P=0.30
Standard Error of Log RR

Supplement: Additional file 1 — ppt. Begg's funnel plot with pseudo 95% confidence limits for all studies with sputum cultures at two to three months. [file 1741-7015-9-81-S1.PPT]

## Slide 1
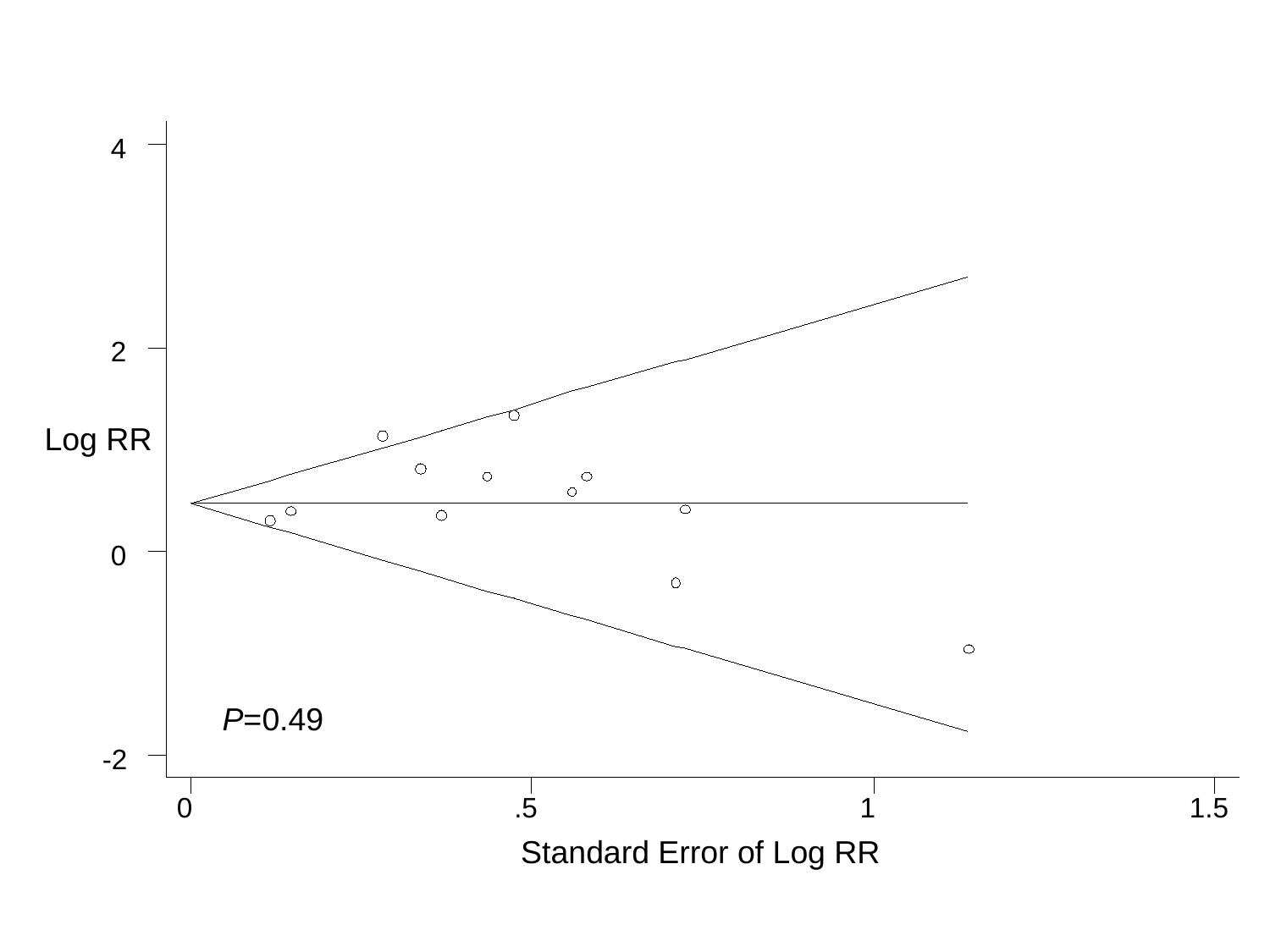

4
2
0
-2
0
.5
1
1.5
Log RR
P=0.49
Standard Error of Log RR

Supplement: Additional file 2 — ppt. Begg's funnel plot with pseudo 95% confidence limits for all studies with the combined outcome of failure and death. [file 1741-7015-9-81-S2.PPT]

## Slide 1
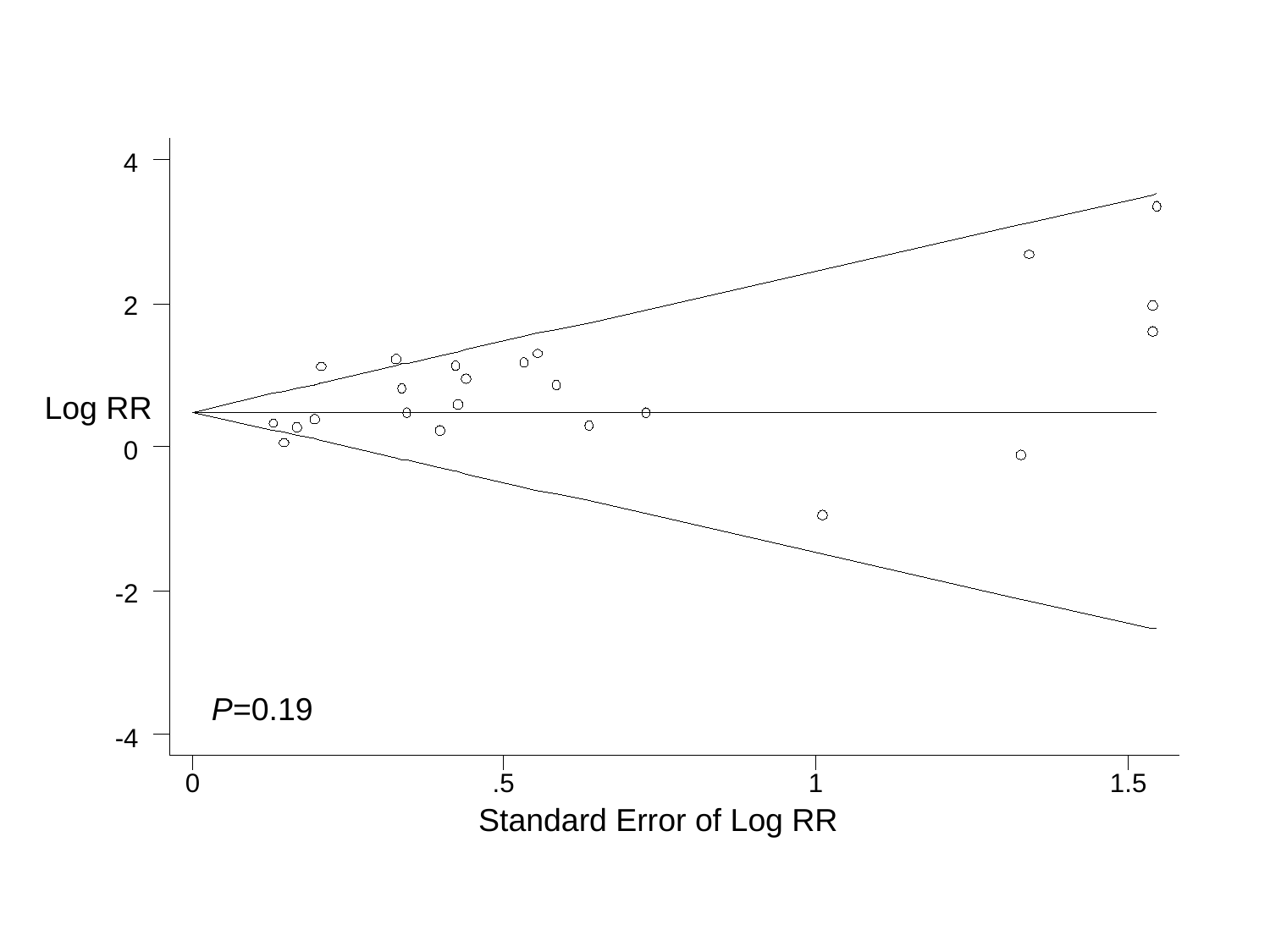

4
2
0
-2
-4
0
.5
1
1.5
Log RR
P=0.19
P=0.19
Standard Error of Log RR

Supplement: Additional file 3 — ppt. Begg's funnel plot with pseudo 95% confidence limits for all studies with the outcome of death. [file 1741-7015-9-81-S3.PPT]

## Slide 1
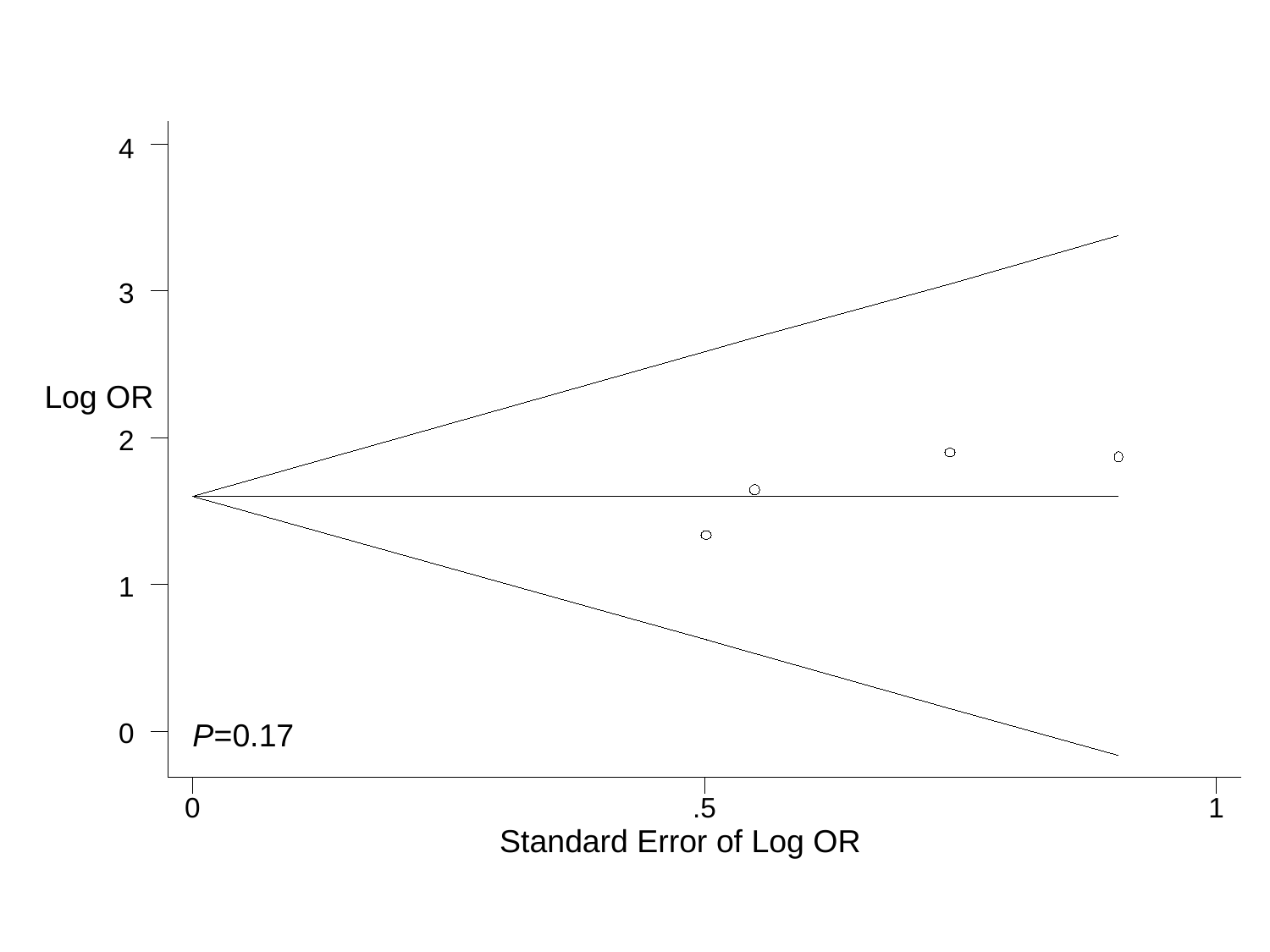

4
3
2
1
0
0
.5
1
Log OR
P=0.17
P=0.17
Standard Error of Log OR

Supplement: Additional file 4 — ppt. Begg's funnel plot with pseudo 95% confidence limits for all studies with the outcome of death adjusted for age and other confounding factors. [file 1741-7015-9-81-S4.PPT]

## Slide 1
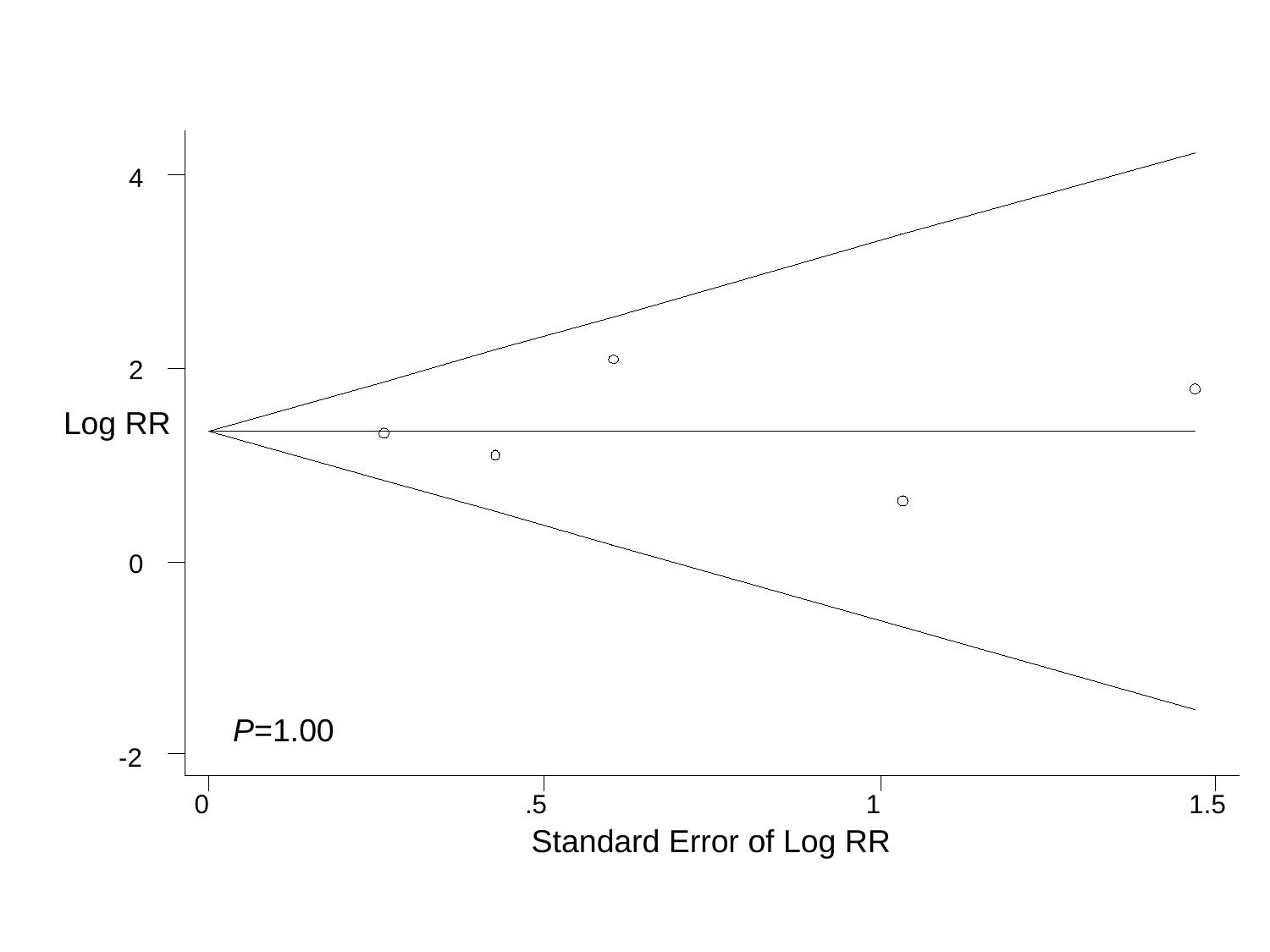

4
2
0
-2
0
.5
1
1.5
Log RR
P=1.00
P=1.00
Standard Error of Log RR

Supplement: Additional file 5 — ppt. Begg's funnel plot with pseudo 95% confidence limits for all studies with TB relapse. [file 1741-7015-9-81-S5.PPT]

## Slide 1
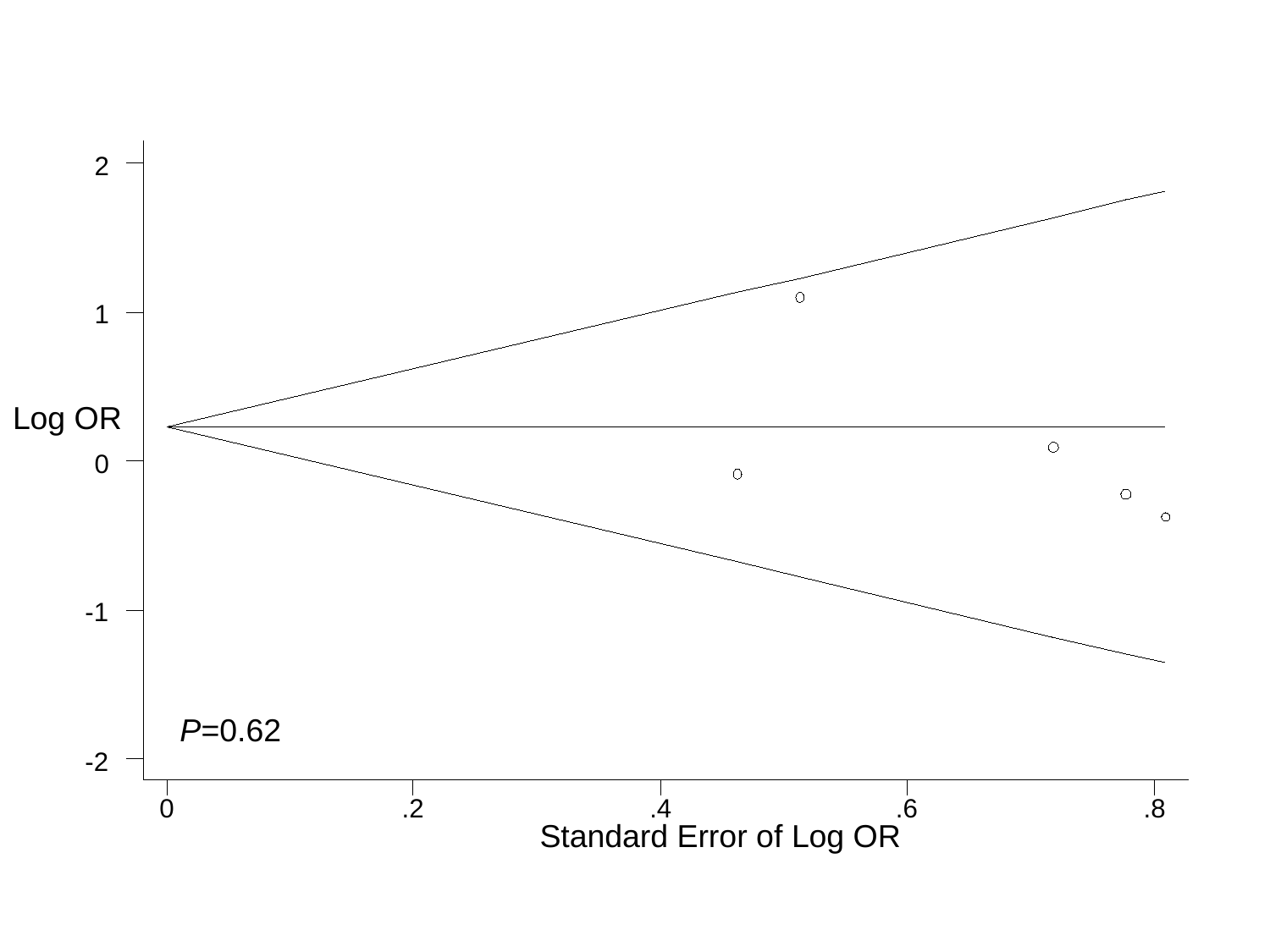

2
1
0
-1
-2
0
.2
.4
.6
.8
Log OR
P=0.62
P=0.62
Standard Error of Log OR

Supplement: Additional file 6 — ppt. Begg's funnel plot with pseudo 95% confidence limits for all studies with recurrent TB that is DR. [file 1741-7015-9-81-S6.PPT]
